# Management of an extensive form of verrucous hemangioma with staged skin grafting: A case report

**DOI:** 10.1002/ccr3.6724

**Published:** 2022-12-09

**Authors:** Samit Sharma, Aadesh Rayamajhi, Abhishek Chapagain, Jayan Man Shrestha

**Affiliations:** ^1^ Department of Plastic Surgery and Burns Tribhuvan University Teaching Hospital Kathmandu Nepal; ^2^ Maharajgunj Medical Campus Tribhuvan University, Institute of Medicine Kathmandu Nepal

**Keywords:** skin graft, venous malformation, verrucous hemangioma

## Abstract

A 20‐year‐old male presented with large and extensive lesions of verrucous hemangioma involving the left lower extremity. The lesions were excised in the supra‐fascial plane. After 10 days, the split‐thickness skin graft was applied over the raw area resulting in good graft take.

## Graphical abstract

Surgical excision of large and extensive verrucous hemangiomas of the limbs in the supra‐fascial plane followed by split‐thickness skin grafting after the development of granulations can result in acceptable cosmetic and functional outcomes.

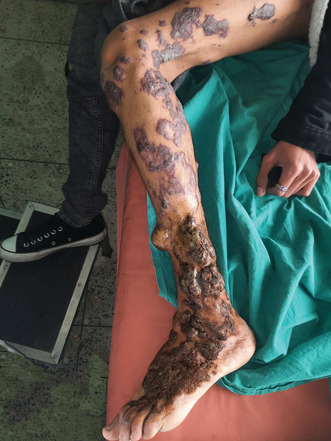

## 1 INTRODUCTION

Verrucous hemangioma (VH) is an uncommon vascular anomaly presenting as reddish‐brown, warty papules, or plaques usually in a linear or serpiginous fashion on the extremities.^1^, ^2^ It begins as a bluish‐red lesion at birth or in early childhood and eventually acquire a verrucous or warty surface. The lesions are typically unilateral and are frequently seen on the extremities and gluteal region. Very rarely they can occur in the trunk^3^ and occur bilaterally.^4^

The definitive diagnosis of VH is made by histopathological examination with clinical correlation. Histologically, VH shows hyperkeratosis, irregular acanthosis, papillomatosis, and vascular proliferation in superficial dermis, deep dermis, and subcutaneous tissue.^5^ VHs do not undergo spontaneous involution, hence early diagnosis and treatment with a suitable modality gives better cosmetic outcome. Cryosurgery, electrocautery, and laser therapy have been used for small lesions.^6^ For large and extensive lesions, surgical excision, or combination of the above modalities has been effective.^7^, ^8^

Herein, we report a case of a 20‐year‐old male with large extensive lesions involving left lower extremity managed with excision and staged split‐thickness skin grafting (STSG).

## 2 CASE PRESENTATION

A 20‐year‐old male from Kathmandu, Nepal presented to the plastic surgery clinic with extensive, warty, brownish lesions, or plaques involving the dorsum of foot, lateral aspect of the lower leg, lateral and posterior aspects of upper leg, and lateral thigh. The lesions were present since birth and enlarged gradually. The lesions were painless to begin with and sometimes bled with trivial trauma. The patient was mainly concerned about cosmesis and frequent bleeding episodes.

**FIGURE 1:**
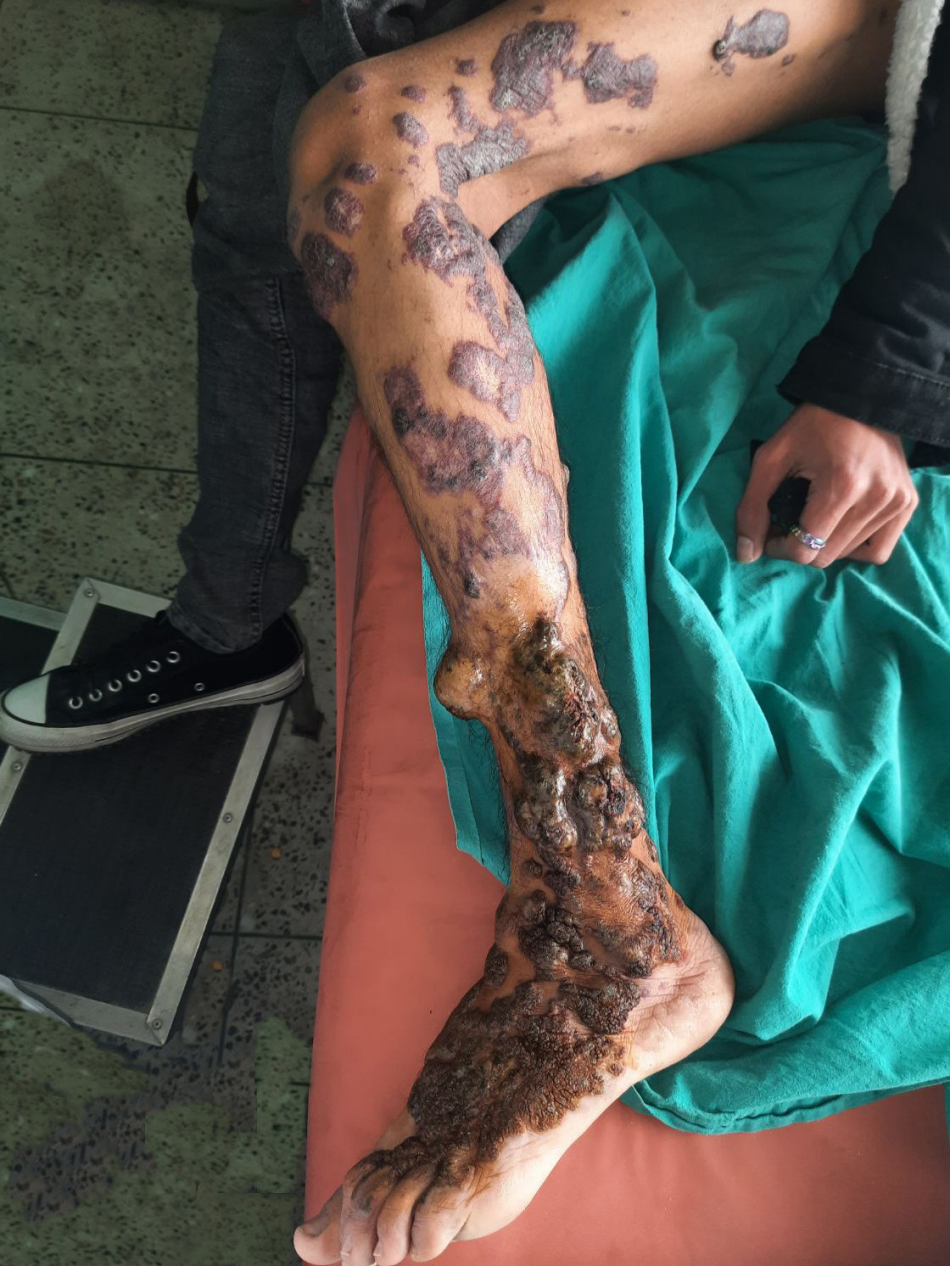
The lesions of verrucous hemangioma seen in the clinic.

**FIGURE 2:**
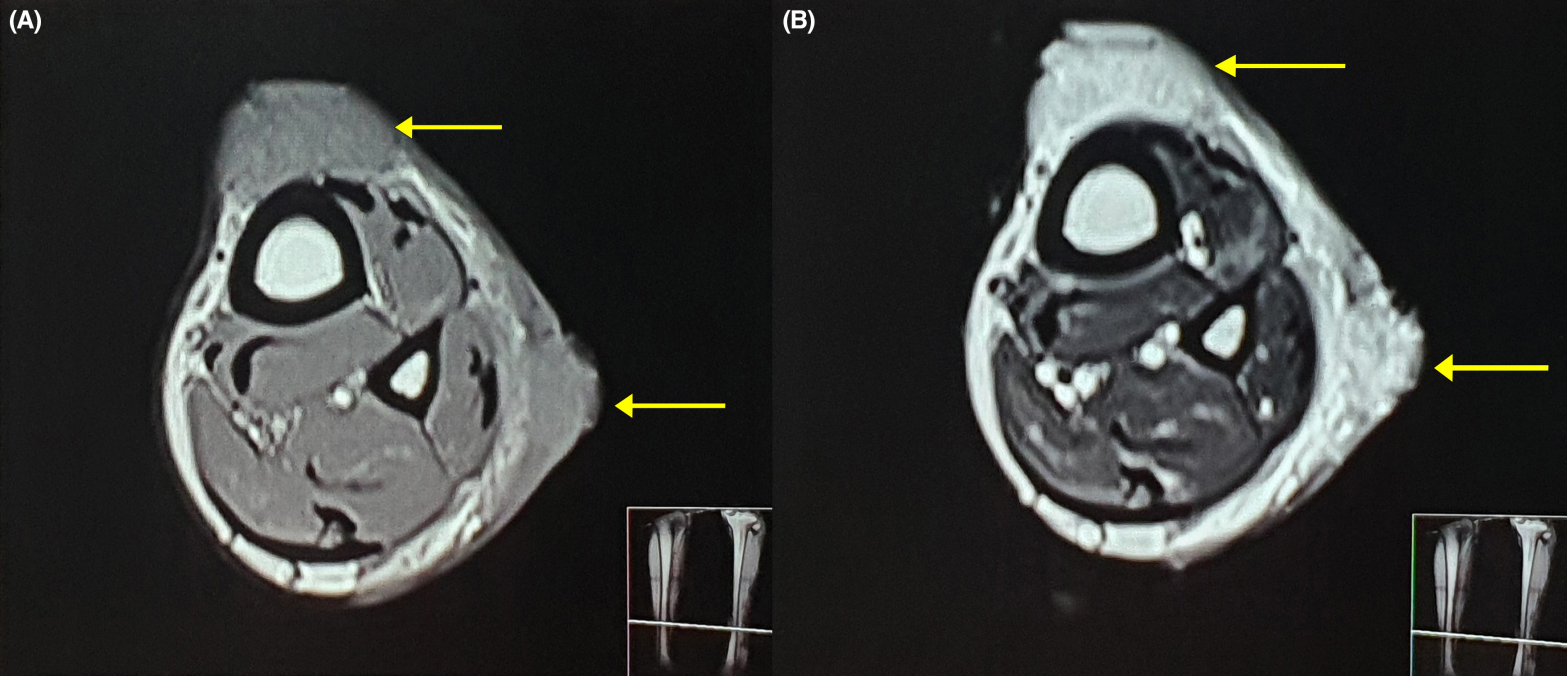
Magnetic resonance imaging of the lesion showing. (A) Iso signal intensity areas (arrows) in the subcutaneous plane on T1 weighted image of left lower leg on axial section. (B) Corresponding high signal intensity areas (arrows) in the subcutaneous plane on T2 weighted image of left lower leg on axial section.

Examination of the plaques revealed raised verrucous areas of red/violet discoloration with hemorrhagic crusts on the lower leg and foot lesions (Figure 1). Moreover, extension contracture could be seen at the base of middle three toes due to the disease process. Small satellite lesions were also seen in the periphery of larger, diffuse lesions. Lesions were non‐tender, non‐compressible, and had no pulsations. Bruits were not heard over the lesions and regional lymph nodes were not enlarged. The right lower extremity was normal and similar lesions were not found elsewhere. Magnetic Resonance Imaging (MRI) of the left lower limb revealed multiple T1 iso and T2 high signal intensity lesions of variable sizes in the subcutaneous plane without extension into muscular plane (Figure 2).

**FIGURE 3:**
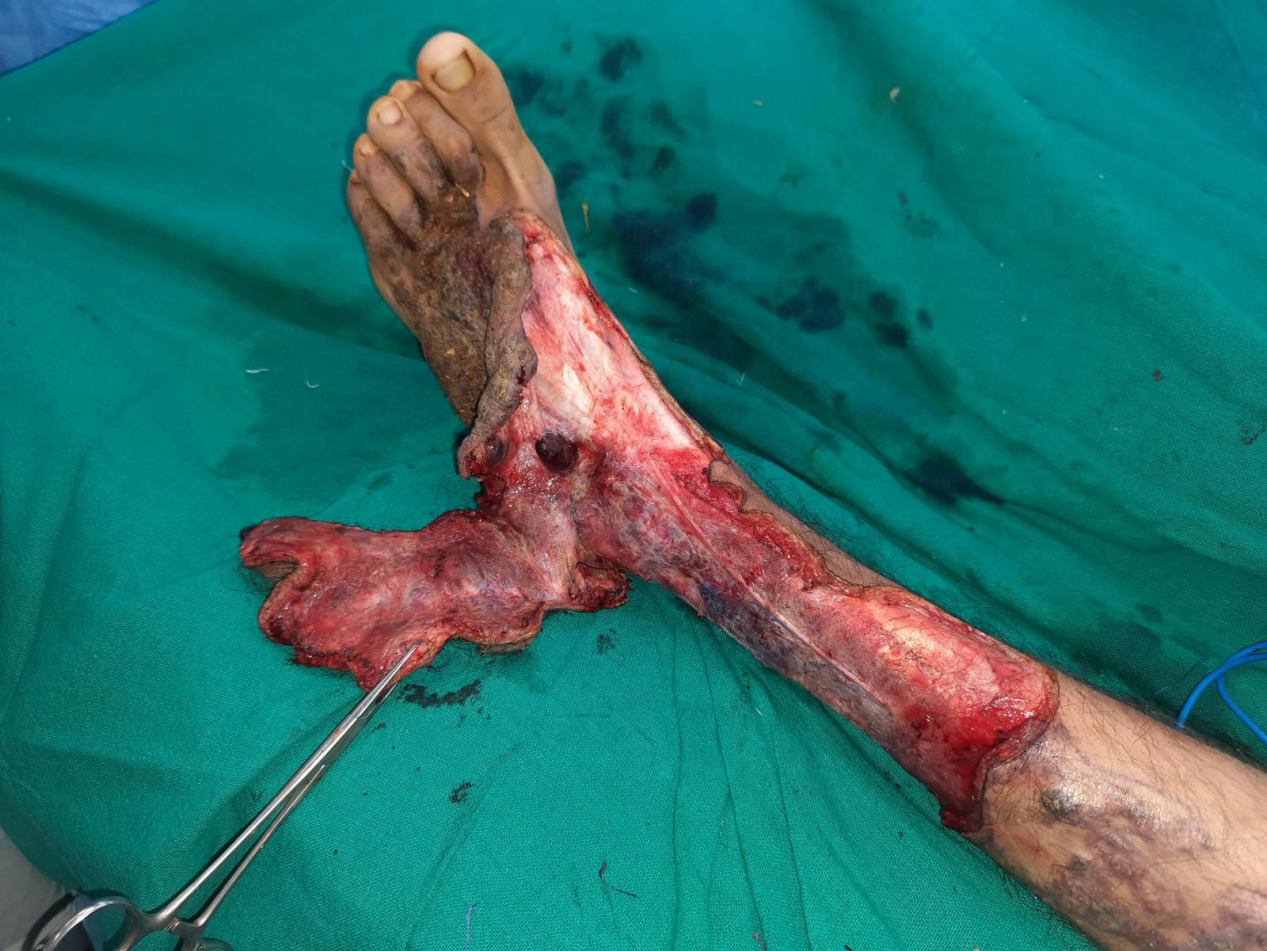
Excision of the lesion in the supra‐fascial plane.

**FIGURE 4:**
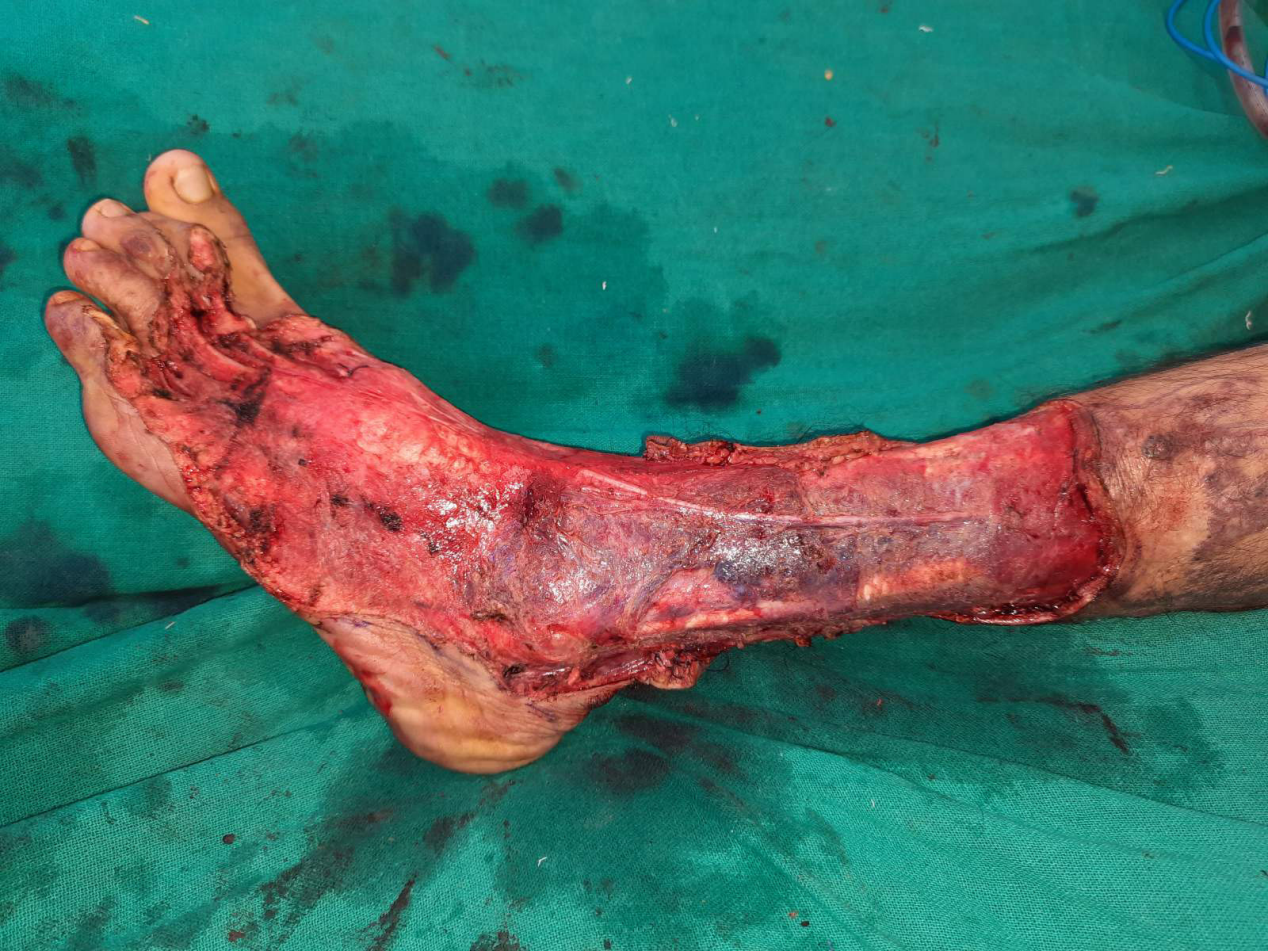
Raw area after excision of verrucous hemangioma.

**FIGURE 5:**
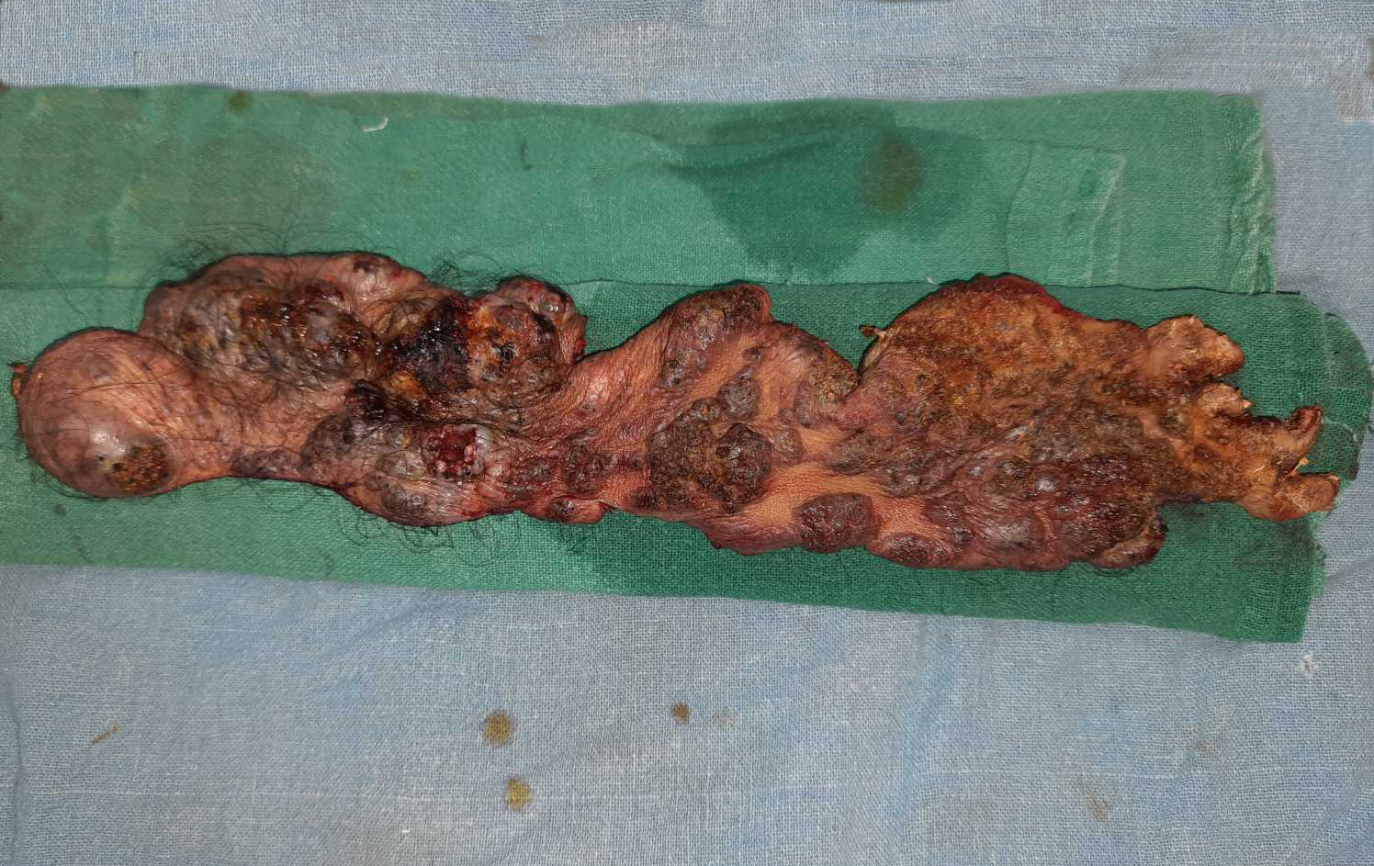
Resected specimen.

**FIGURE 6:**
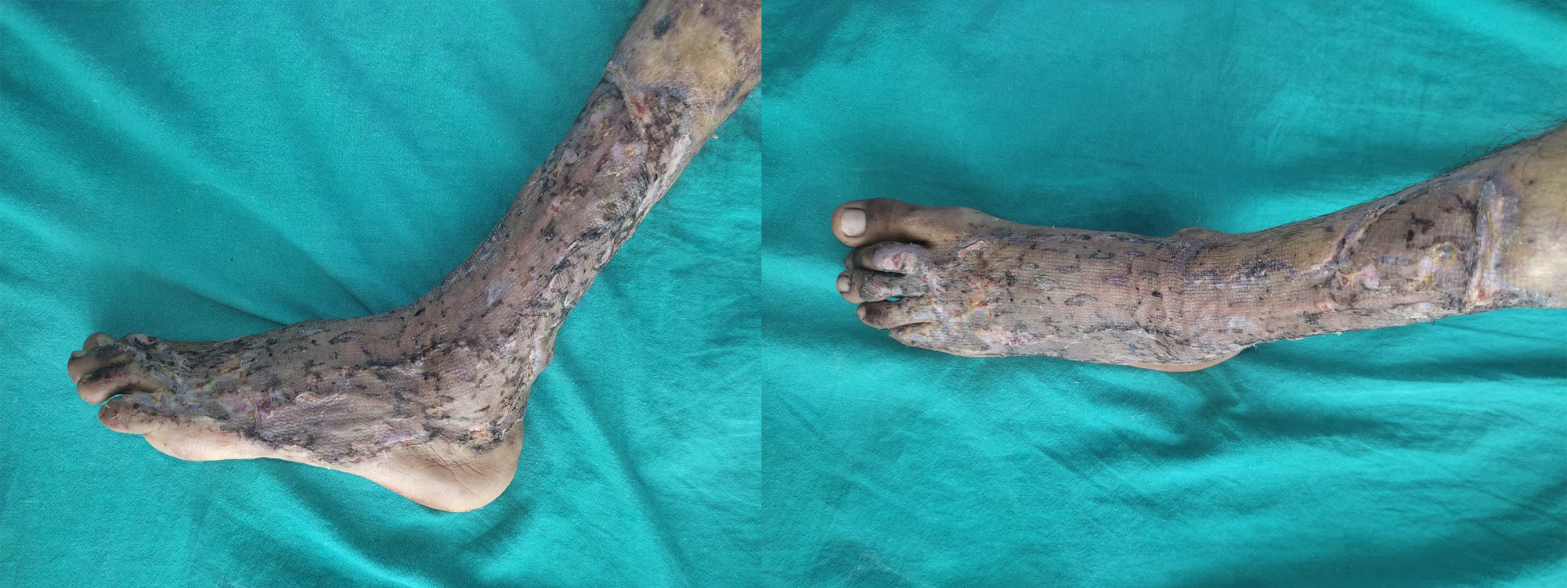
Two‐week postoperative picture; good skin graft takes 2 weeks after skin grafting.

**FIGURE 7:**
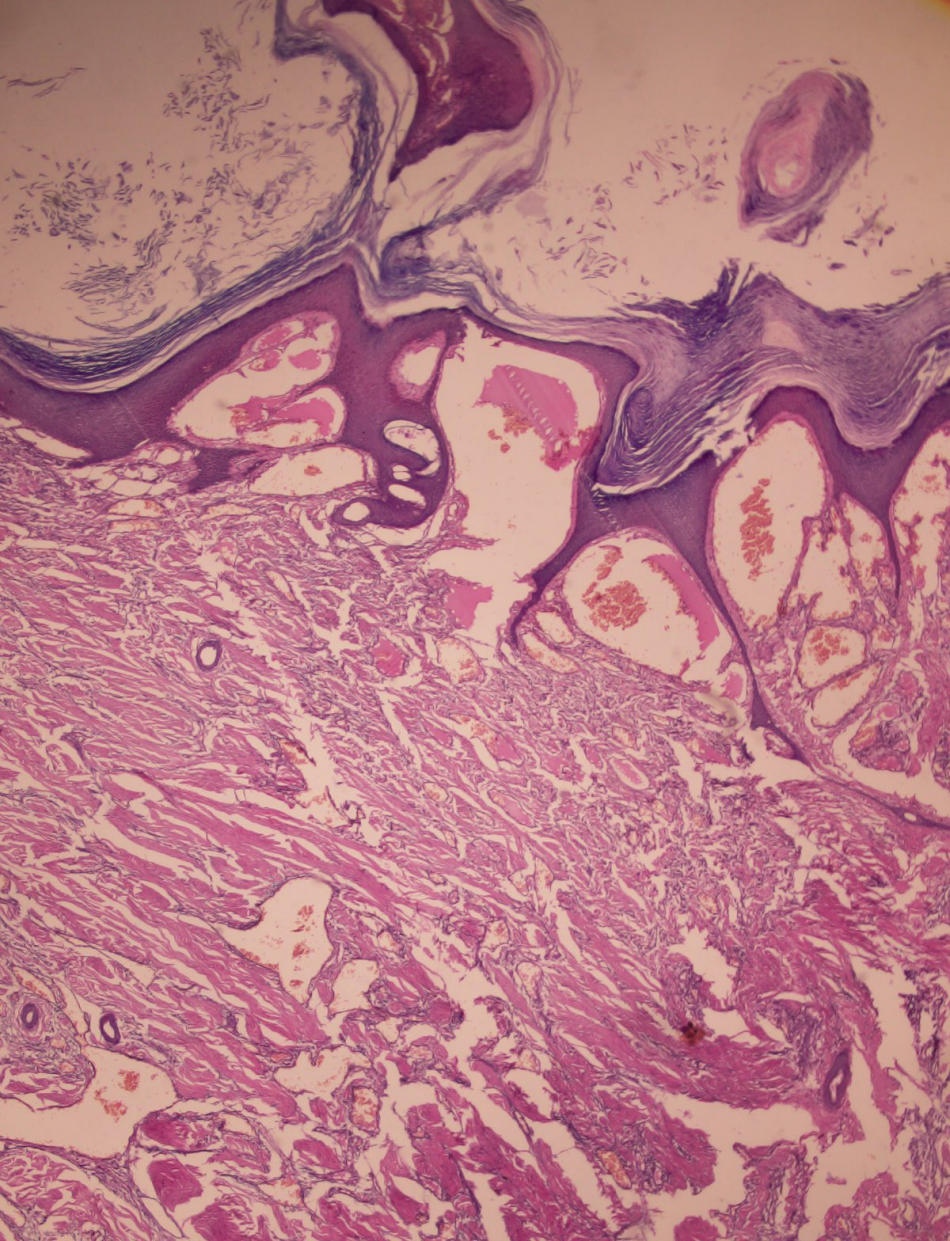
Histopathology of the specimen.

The surgery of the verrucous hemangioma was performed in two stages: the first stage consisted of excision under tourniquet control and the second stage, split‐thickness skin grafting. Intraoperatively deep infiltration of the tissue planes involving the skin, subcutaneous, and deeper tissues was seen. The excision was carried out in supra‐fascial plane (Figures 3, 4, 5). After the first stage, alternate‐day dressings were done until the wound was granulated thoroughly. After 10 days, a split‐thickness split graft was harvested from the right thigh and applied on the post‐excision raw areas. The first dressing of the skin graft was done on the fourth day, then every 2 days. The graft take was good with all the wounds healed in 2 weeks and with the good aesthetic outcome (Figure 6). Histopathology examination of the specimen revealed irregular papillomatosis, acanthosis, and hyperkeratosis of the epidermis with multiple, thin‐walled, dilated blood‐filled spaces in the dermis (Figure 7).

## 3 DISCUSSION

Verrucous hemangioma (VH) is a rare congenital vascular anomaly. The term VH was coined by Halter in 1937 who reported a 16‐year‐old boy with a typical lesion.^9^ In 1967, Imperial and Helwig defined VH as a congenital vascular malformation comprising capillary or cavernous hemangioma in the dermis and subcutaneous tissue associated with reactive epidermal acanthosis, papillomatosis, and hyperkeratosis, distinguishing it from angiokeratoma.^10^ Various studies have described similar lesions using various names such as angiokeratoma circumscriptum naeviforme,^11^ angiokeratoma circumscriptum,^12^ keratotic hemangioma,^13^ and verrucous venous hemangioma.^14^ According to International Society for the Study of Vascular Anomalies (ISSVA), vascular anomalies can be classified into tumors (endothelial cell proliferation) or malformations (structural or morphologic anomalies resulting from faulty embryonic morphogenesis).^14^ Some authors consider VH as a vascular tumor^2^ whereas the others as a malformation.^8^, ^14^, ^15^ Thus, classification of VH is still unclear because it shows clinical features similar to those of vascular malformation but immunoprofile similar to vascular neoplasms. However, the ISSVA 2018 classification places verrucous hemangioma under venous malformations.^14^

Though most studies have discussed the classification and histopathology of VMs, only a few have discussed the surgical management of extensive form of the disease. Minimally invasive procedures such as cryotherapy, electrocautery, and laser have been used for smaller lesions.^6^ Because of infiltration of the subcutaneous tissue and sometimes deeper, surgery appears to be the best treatment for VH. The excision in our case, was carried out in the supra‐fascial plane taking care to preserve the layers of fascia and paratenon which would provide a vascular bed for split‐thickness skin grafting. Deeper plane could result in better clearance of the lesion but would lead to the exposure of long tendons of the distal leg and foot posing a great challenge for reconstruction. Very large flaps possibly free flaps would be required for the coverage of the exposed structures. Small lesions can be treated with simple excision or serial excision, however larger areas require a skin graft or flap for coverage. In our case, the post‐excision area was too large to be covered by a flap or a full‐thickness skin graft, hence split‐thickness skin graft was used.

**FIGURE 8:**
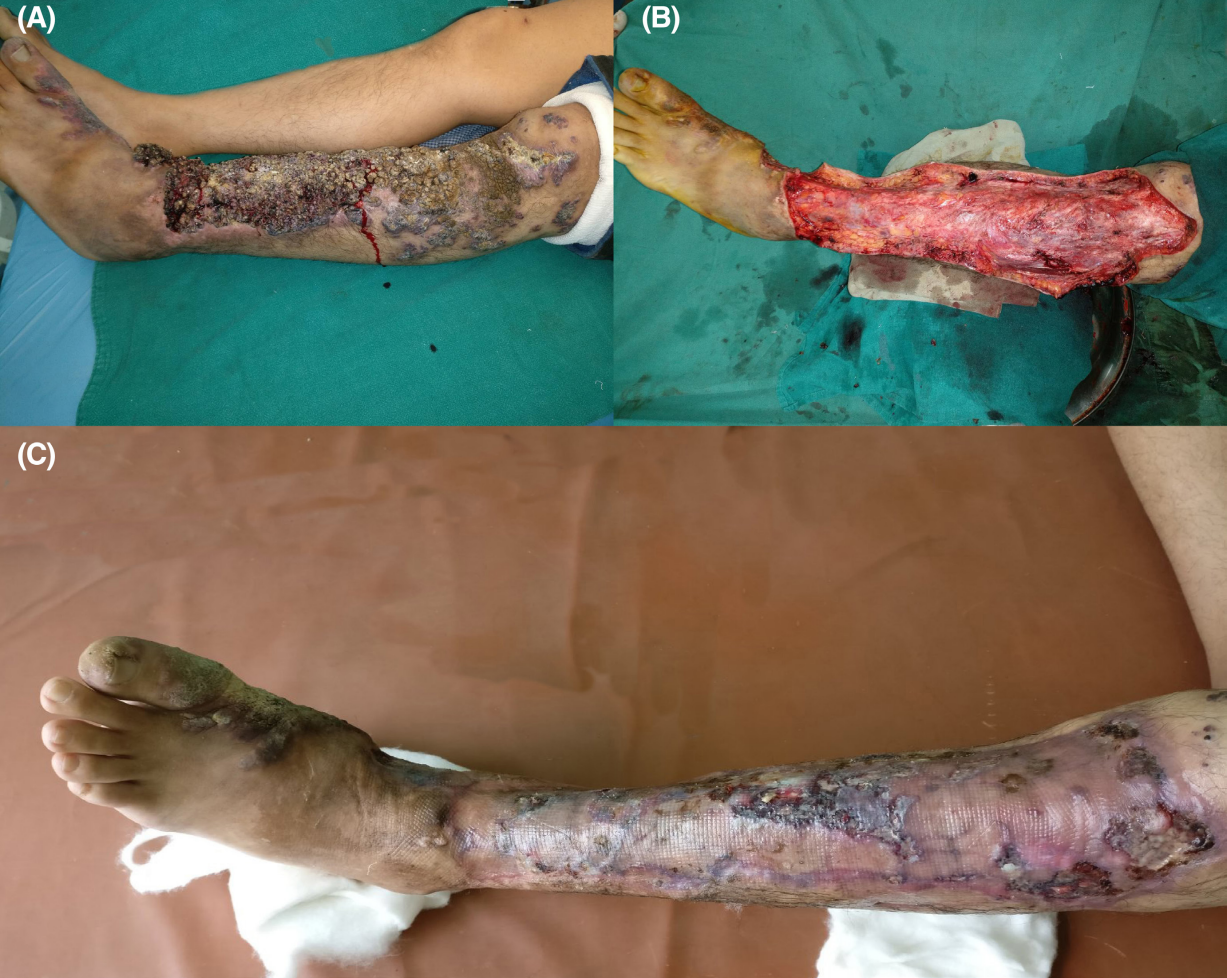
A similar case operated 3 years back: (A) Preoperative picture of the lesion. (B) After the excision of the lesion. (C) Skin graft “take” after two sessions of re‐grafting following partial graft loss.

The surgical treatment was carried out in two stages. Three years prior, a similar case was operated with excision and skin grafting in the same setting, however, there was partial graft loss and the area had to be re‐grafted two times (Figure 8). The graft loss could be due to hematoma from the leaky capillaries or vessels of the lesion or due to residual infiltrative disease. Hence, this time, the surgery was planned to be carried out in two stages, excision in the first stage and skin grafting in the second. As most of the large vessels seen during the dissection were ligated and small ones cauterized, the gap between the first and the second probably helped in sloughing of the residual disease if any, and in the growth of an optimally vascularized bed of granulation tissue for skin grafting. Skin grafting over this bed resulted in better skin graft take and better cosmesis compared to the one done in the single setting. Moreover, the areas where small areas of tendon were exposed such as the bases of toes in the forefoot, which are difficult areas to cover, were also covered with granulations. The excision at the bases of the toes also resulted in the release of the contracture that was present due to the disease process and the area was covered with a skin graft resulting in a better functional outcome.

No recurrence has been detected in the second case where skin grafting was done in the same setting. Also, no recurrence or any complication has been reported up to 3 months follow‐up of the case. However, further follow‐up is required to see for recurrence. Given the patient's satisfaction with excision and staged skin grafting, we would recommend this modality of treatment for very large and extensive VHs.

## 4 CONCLUSION

Large and extensive verrucous hemangiomas of the limbs mandate excision which when carried out in two stages, first excision, and then skin grafting over the granulated wound can result in better skin graft take with satisfactory cosmetic and functional outcomes.

## AUTHOR CONTRIBUTIONS

SS and AR: involved in concept, collecting information, manuscript writing, and participated in literature review and edited the draft. SS and JMS: involved in patient care team and also independently reviewed the manuscript. SS, AR, AC, and JMS: re‐edited the draft and reshaped it into this manuscript. All authors approved the final version of the manuscript and agree to be accountable for all aspects of the work in ensuring that questions related to the accuracy or integrity of any part of the work are appropriately investigated and resolved.

## CONFLICT OF INTEREST

The authors declare that there is no conflict of interest regarding the publication of this paper.

## ETHICAL APPROVAL

Need for ethical approval waived. Consent from the patient is deemed to be enough.

## CONSENT

Written informed consent was obtained from the patient for publication of this case report and any accompanying images. A copy of the written consent will be available for review if asked by the editor‐in‐chief of this journal.

## Data Availability

Not applicable.
